# Impact of removing OPTN region from vascularized composite allograft allocation

**DOI:** 10.3389/frtra.2024.1399357

**Published:** 2024-05-16

**Authors:** Sarah E. Booker, Jesse Howell, Thomas G. Dolan, Kelley Poff, Krissy Laurie, Wida S. Cherikh, David K. Klassen, Jennifer L. Wainright

**Affiliations:** ^1^ Research Department, United Network for Organ Sharing, Richmond, VA, United States; ^2^ Policy and Community Relations Department, United Network for Organ Sharing, Richmond, VA, United States; ^3^ Member Quality Department, United Network for Organ Sharing, Richmond, VA, United States; ^4^ Office of the Chief Medical Officer, United Network for Organ Sharing, Richmond, VA, United States

**Keywords:** vascularized composite allograft, VCA transplantation, organ allocation, allocation policy, policy evaluation

## Abstract

On 6/18/2020, the Organ Procurement and Transplantation Network (OPTN) implemented new policy replacing OPTN region with a 500 nautical mile (NM) circle around the donor hospital for the purpose of vascularized composite allograft (VCA) allocation. We used OPTN data to assess deceased donor VCA transplants in the 3 years pre- (6/19/2017–6/17/2020) vs. post-implementation (6/18/2020–6/17/2023). A total of 19 deceased donor VCA transplants were performed pre-policy (10 uterus, 3 bilateral upper limb, 1 unilateral upper limb, 3 face, 1 abdominal wall and 1 penis), and 11 post-policy (4 uterus, 1 bilateral upper limb, 2 face, 1 trachea, 2 abdominal wall, and 1 bilateral upper limb and face). Median distance from donor hospital to transplant hospital increased from 70 NM (range: 0–524 NM) pre-policy to 119 NM (range: 0–464 NM) post-policy. The majority of transplants in both policy eras were within 500 NM of the donor hospital [89.5% (*N* = 17/19) vs. 100% (*N* = 11/11)] and most remained within the same OPTN region as the donor hospital [68.4% (*N* = 13/19) vs. 90.9% (*N* = 10/11)]. Although it is difficult to draw strong conclusions about the policy's impact due to the low transplant volume and timing of implementation relative to the COVID-19 pandemic, data in the 3 years post-implementation suggest that 500 NM circles were a reasonable replacement for OPTN region in VCA allocation. The OPTN will continue to review data to monitor the policy's impact and inform future changes to VCA allocation, such as the transition to continuous distribution, a points-based framework expected to replace the current framework.

## 1 Introduction

Vascularized composite allograft (VCA) transplantation encompasses a diverse group of organs that meet the 9 criteria for VCA outlined in the Organ Procurement and Transplantation Network (OPTN) Final Rule (1). VCA is still a relatively new and evolving field of transplantation, with the first successful VCA transplants performed in the late 1990′s (2–4). To date, VCA transplants performed in the U.S. have included upper limb, face, scalp, larynx, trachea, abdominal wall, penis, and uterus (5, 6).

On 3 July 2014, the OPTN was granted oversight of VCA allocation and transplantation within the U.S., and the OPTN VCA Transplantation Committee developed an initial set of policies for VCA, including policy for allocation (1, 7). This initial allocation policy required organ procurement organizations (OPOs) to offer deceased donor VCAs to candidates listed at transplant hospitals within the same OPTN region as the donor hospital before offering to candidates outside the region. OPTN regions are administrative boundaries consisting of groups of donation service areas (DSA, a geographic area that is served by one OPO). There are currently 11 OPTN regions, which vary in both population size and geographic area (8). At the time of the initial VCA allocation policy development, DSA and OPTN region were also used as geographic units of allocation for other solid organs. While other allocation policies used DSA as the first unit of allocation, the original VCA allocation policy used OPTN region as the first unit of allocation to facilitate sharing of VCAs as broadly as feasible within the constraints of cold ischemic time (CIT) (7). VCA candidates were ranked by waiting time within their respective classifications (inside/outside region).

In 2018, the U.S. Department of Health and Human Services determined that the use of DSA and OPTN region in organ allocation policies could not be justified under the OPTN Final Rule, as these are administrative boundaries that were not designed for the purpose of allocation (9). The OPTN Board of Directors directed the OPTN VCA Transplantation Committee to develop new policy removing OPTN region from VCA allocation. The Committee considered several policy options and ultimately recommended replacing OPTN region with a 500 nautical mile (NM) circle around the donor hospital. Consistent with the rationale for the original VCA allocation policy, the selection of the 500 NM circle size was informed by the desire to allocate VCAs over as broad a geographic area as possible without incurring unacceptably long CIT that could negatively impact post-transplant outcomes or result in organ non-use. This new VCA allocation policy was approved by the OPTN Board of Directors in June 2019 and implemented on 18 June 2020 (10, 11).

Post-implementation evaluation of changes to organ allocation policy is important to assess whether the policy has met its objectives, monitor for any unintended consequences, and identify areas for further improvement. The purpose of this analysis is to assess the impact of the current VCA allocation policy in the 3 years since implementation.

## 2 Methods

### 2.1 Cohort

The cohort for this analysis included all VCA transplants performed in the U.S. between 1 January 1998 and 17 June 2023. Pre- and post-policy comparisons were limited to deceased donor VCA transplants in the 3 years post-policy (18 June 2020–17 June 2023), compared with the 3 years immediately prior to implementation (19 June 2017–17 June 2020).

### 2.2 Data source

This study used data from the Organ Procurement and Transplantation Network. The OPTN data system includes data on all donors, wait-listed candidates, and transplant recipients in the U.S., submitted by the members of the OPTN. The Health Resources and Services Administration (HRSA), U.S. Department of Health and Human Services provides oversight to the activities of the OPTN contractor. VCA transplant programs submit information about their candidates and recipients on VCA Transplant Candidate Registration (TCR) forms and Transplant Recipient Registration (TRR) forms, respectively. Institutional review board (IRB) exemption was obtained from HRSA.

### 2.3 Statistical analysis

We performed a descriptive analysis of VCA transplants pre- vs. post-policy, including comparisons of recipient characteristics [age, race/ethnicity, birth sex, calculated panel reactive antibody (CPRA), blood type], distance from donor hospital to transplant hospital, share type, CIT, and time from listing to transplant. For recipients of multiple VCA grafts, including bilateral upper limb and combined bilateral upper limb and face, CIT is the highest CIT value reported for the recipient's grafts. Statistical comparisons used Fisher's exact test for categorical variables and the Kruskal–Wallis test for continuous variables. Ability to detect statistical significance was limited by the low volume of VCA transplants.

## 3 Results

**Figure 1:**
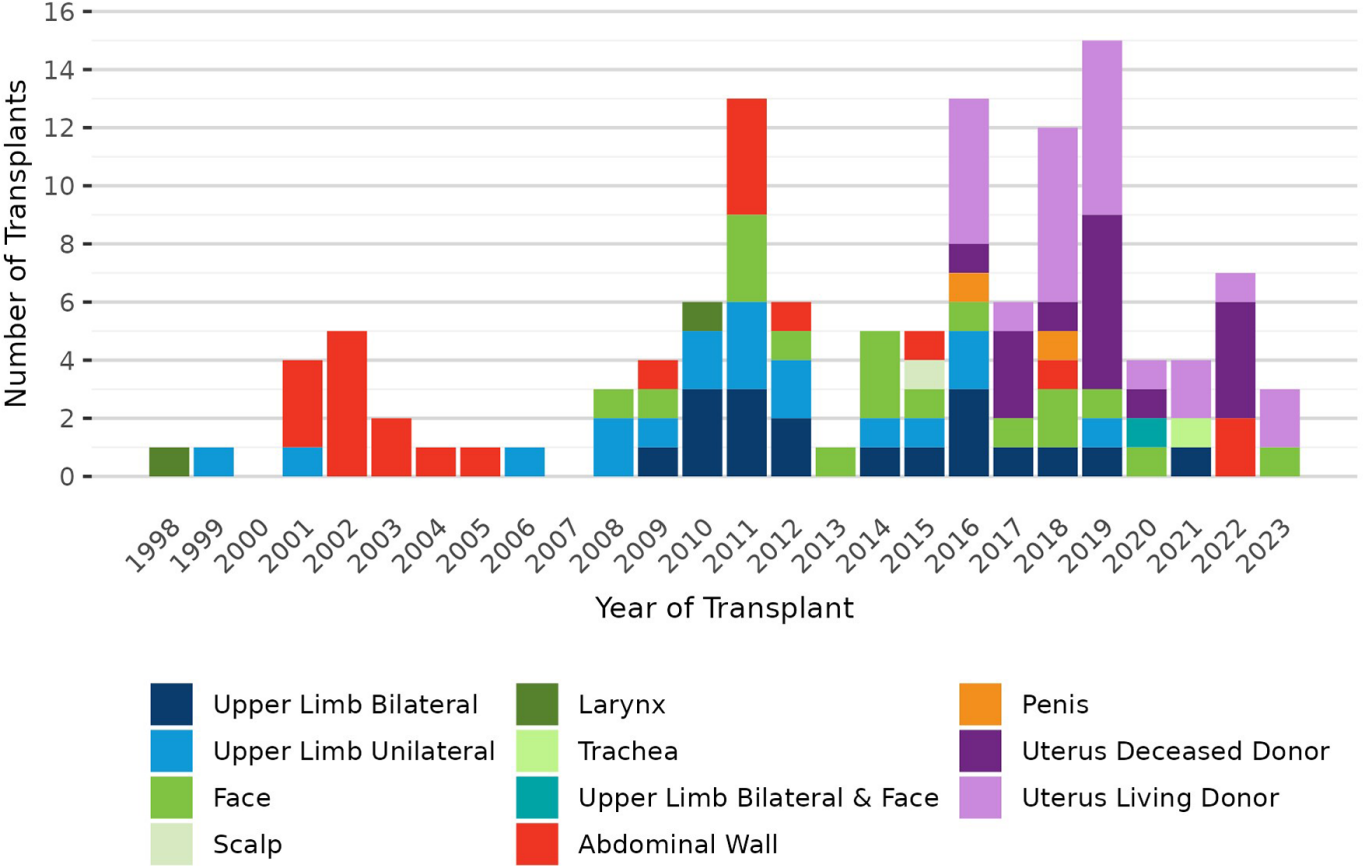
Number of VCA transplants in the U.S. by year and VCA type, 1 January 1998–17 June 2023.

**Figure 2:**
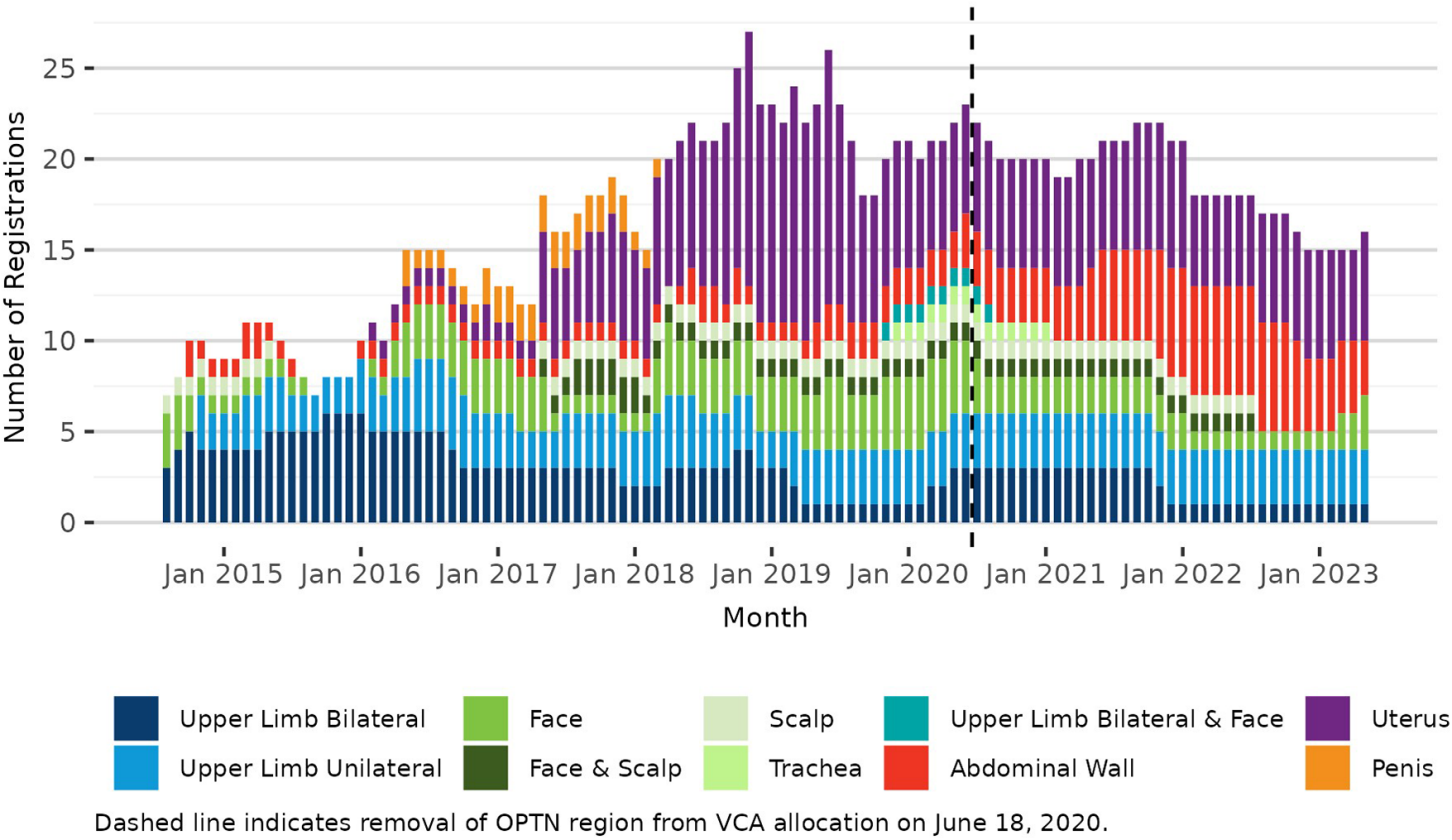
Number of VCA registrations on the OPTN waiting list on the first day of each month, 3 July 2014–17 June 2023.

Pre-policy, 7 transplant hospitals performed a total of 19 deceased donor VCA transplants (10 uterus, 3 bilateral upper limb, 1 unilateral upper limb, 3 face, 1 abdominal wall, and 1 penis); post-policy, 7 transplant hospitals performed a total of 11 deceased donor transplants (4 uterus, 1 bilateral upper limb, 2 face, 1 trachea, 2 abdominal wall, and 1 combined bilateral upper limb and face). It is important to note that this change in VCA allocation was implemented 3 months after the onset of the COVID-19 pandemic in March 2020. VCA transplant volumes declined in 2020 with the pandemic's onset and have not recovered to pre-pandemic volumes (Figure 1). In contrast, there was little change in the volume or composition of the VCA waiting list after policy implementation or COVID-19 onset (Figure 2).

The majority of VCA transplant recipients in both policy eras were White, non-Hispanic (89.5% pre-policy vs. 81.8% post-policy) with low sensitization (CPRA 0%–19%: 78.9% vs. 54.5%) (Table 1). Median age was similar at 34 years [interquartile range (IQR): 32–42 years] pre-policy and 32 years (IQR: 25–42 years) post-policy. Pre-policy, the majority of recipients had blood type O (52.6%), while the majority of recipients post-policy had blood type A (63.6%). All uterus recipients were female (*N* = 10 pre-policy vs. *N* = 4 post-policy); most non-uterus recipients pre-policy were male (77.8%, *N* = 7/9), while the majority of non-uterus recipients post-policy were female (57.1%, *N* = 4/7).

**Table 1:** VCA transplant recipient characteristics by policy era.

	Pre-policy *N* (%)	Post-policy *N* (%)	*p*-value
*N*	19	11	
VCA type			0.63
Uterus	10 (52.6)	4 (36.4)	
Upper limb	4 (21.1)	1 (9.1)	
Face	3 (15.8)	2 (18.2)	
Trachea	0 (0.0)	1 (9.1)	
Abdominal wall	1 (5.3)	2 (18.2)	
Penis	1 (5.3)	0 (0.0)	
Upper limb and face	0 (0.0)	1 (9.1)	
Median age, years (IQR)	34 (32–42)	32 (25–42)	0.22
Age group			0.92
18–34	10 (52.6)	7 (63.6)	
35–49	5 (26.3)	2 (18.2)	
50–64	3 (15.8)	2 (18.2)	
65+	1 (5.3)	0 (0.0)	
Race/ethnicity			0.24
White, non-Hispanic	17 (89.5)	9 (81.8)	
Black, non-Hispanic	1 (5.3)	0 (0.0)	
Hispanic/Latino	0 (0.0)	2 (18.2)	
Native Hawaiian/Pacific Islander	1 (5.3)	0 (0.0)	
Birth sex			0.70
Female	12 (63.2)	8 (72.7)	
Male	7 (36.8)	3 (27.3)	
CPRA			0.11
0%	13 (68.4)	3 (27.3)	
1%–19%	2 (10.5)	3 (27.3)	
20%–79%	2 (10.5)	1 (9.1)	
80%–100%	0 (0)	0 (0)	
Not reported	2 (10.5)	4 (36.4)	
Blood type			0.10
A	5 (26.3)	7 (63.6)	
B	4 (21.1)	0 (0.0)	
AB	0 (0)	0 (0)	
O	10 (52.6)	4 (36.4)	
Distance from donor hospital			0.52
0–500 NM	17 (89.5)	11 (100.0)	
>500 NM	2 (10.5)	0 (0.0)	
Median distance, NM (range)	70 (0–524)	119 (0–464)	0.67
Share type			0.36
Same region	13 (68.4)	10 (90.9)	
National	6 (31.6)	1 (9.1)	
Median CIT, minutes (range)	292 (0–510)	210 (72–518)	0.75
Missing (*N*)	4	4	
Median days from listing to transplant (IQR)	176 (76–481)	214 (132–278)	0.98

CIT, cold ischemic time; CPRA, calculated panel reactive antibody; IQR, interquartile range; NM, nautical miles.

**Figure 3:**
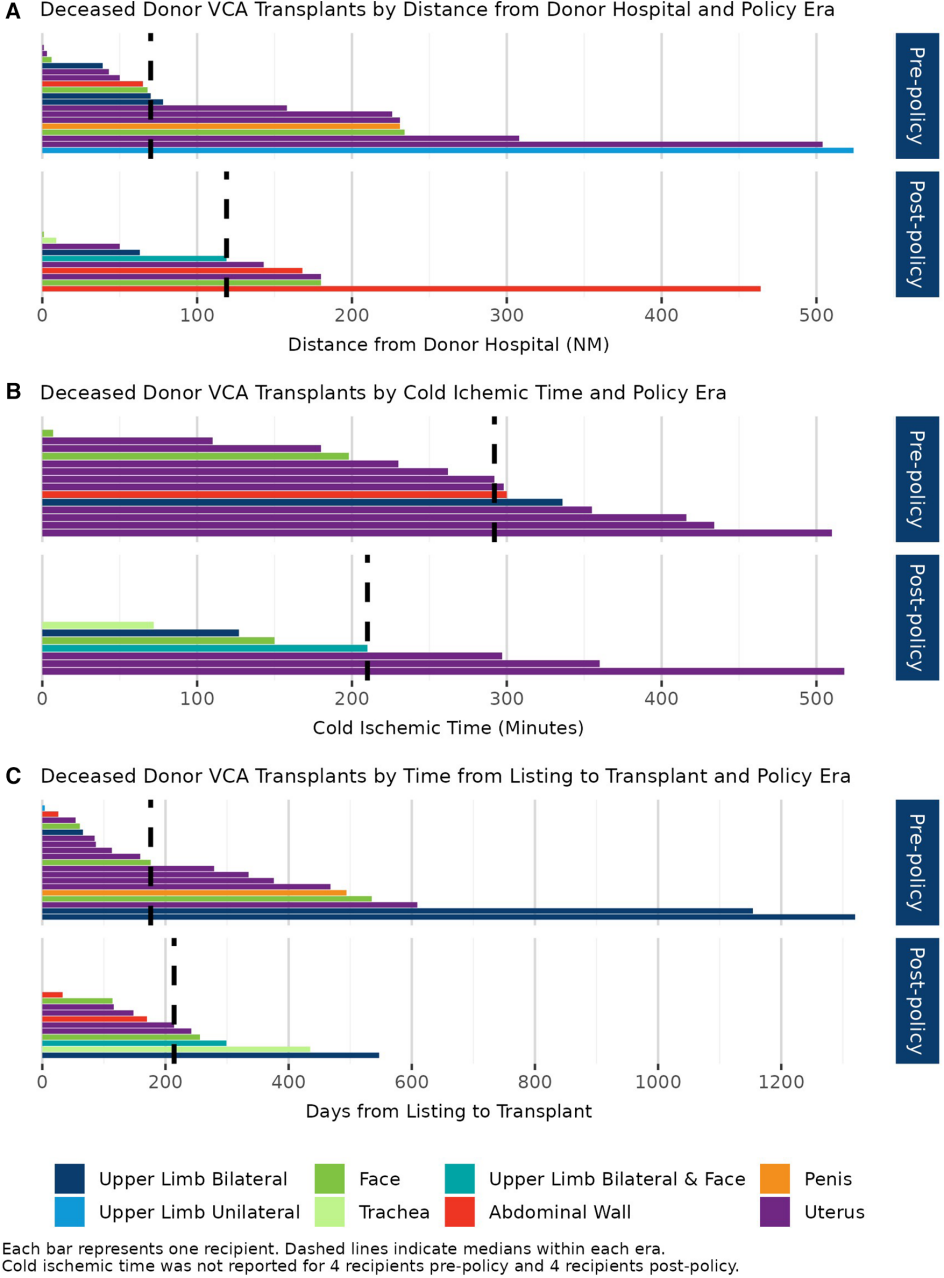
(**A**) Deceased donor VCA transplants by nautical mile (NM) distance from donor hospital to transplant hospital and policy era. (**B**) Deceased donor VCA transplants by cold ischemic time and policy era. (**C**) Deceased donor VCA transplants by time from listing to transplant and policy era. Pre-policy: 19 June 2017–17 June 2020. Post-policy: 18 June 2020–17 June 2023. Each bar represents one recipient. Dashed lines indicate medians within each era.

Median distance from donor hospital to transplant hospital increased from 70 NM (range: 0–524 NM) to 119 NM (range: 0–464 NM) after policy implementation; this increase was not statistically significant (*p* = 0.67) (Figure 3A). Most transplants in both policy eras occurred within 500 NM of the donor hospital [89.5% (*N* = 17/19) vs. 100% (*N* = 11/11)]. The proportion of transplants allocated within the same OPTN region as the donor hospital increased from 68.4% (*N* = 13/19) to 90.9% (*N* = 10/11) (*p* = 0.52). Median CIT decreased from 292 minutes (range: 0–510 min) to 210 min (range: 72–518 min) (*p* = 0.75); CIT was not reported for 4 transplants pre-policy (21.1%) and 4 transplants post-policy (36.4%) (Figure 3B). Median time from listing to transplant for transplant recipients was similar at 176 days (IQR: 76–481 days) pre-policy vs. 214 days (IQR: 132–278 days) post-policy (*p* = 0.98) (Figure 3C).

## 4 Discussion

Replacing OPTN region with a 500 NM circle around the donor hospital brought VCA allocation into alignment with the OPTN Final Rule through the use of a geographic unit that is consistently applied to all candidates regardless of their location of listing. The volume of VCA transplants remained low and decreased after the onset of the COVID-19 pandemic with 11 deceased donor VCA transplants performed in the 3 years after the allocation change, vs. 19 deceased donor transplants in the 3 years prior to the new policy. The low transplant volume and the timing of the policy implementation relative to the COVID-19 pandemic make it difficult to draw strong conclusions about the policy's impact. While median distance from donor hospital to transplant hospital increased by approximately 50 NM post-implementation, most transplants remained within 500 NM of the donor hospital, with 75% occurring within 200 NM. This finding is consistent with data reviewed during the policy development, which showed that most VCA transplants remained within 200 NM of the donor hospital at the time of analysis (9). The majority of transplants were allocated within the same OPTN region as the donor hospital both pre- and post-policy. Median CIT decreased by 1.4 hours (292 vs. 210 min) and maximum CIT was similar at approximately 8.5 h (510 vs. 518 min).

With fewer than 20 VCA candidates on the OPTN waiting list as of 1 January 2024 and no more than 7 candidates waiting for any one specific type of VCA (12), it is unlikely that many candidates waiting for the same VCA organ would be compatible with the same donor after screening for donor-recipient factors (e.g., blood type compatible, histocompatibility, sex, skin tone, size matching). As such, optimizing the order in which candidates receive offers is likely less crucial for VCA than for allocation of other solid organs, where many candidates are a potential match for a given donor. However, as the field of VCA transplantation continues to grow and evolve, demand for VCA may increase, which would increase the importance of the allocation order.

Future changes to VCA allocation include the transition to a continuous distribution framework, a points-based system that is expected to replace the current classification-based system (13–16). In the current framework, VCA candidates are ranked by waiting time within distance-based classifications. Under continuous distribution, candidates are ranked using a composite allocation score intended to simultaneously account for all factors (“attributes”) relevant to allocation. A continuous distribution policy for VCA could be limited to just two attributes based on distance and waiting time, similar to current allocation, or could incorporate additional factors such as sensitization or blood type.

This analysis has several limitations. First, the small number of transplants both pre- and post-policy precludes more robust statistical analyses. Second, this policy was implemented 3 months after the onset of the COVID-19 pandemic, and it is not possible to isolate the impact of the allocation change from that of the pandemic or other factors affecting the field of VCA transplantation. Third, data on CIT should be interpreted with caution. CIT data were missing for 4 recipients in each policy era, or 21.1% of transplants pre-policy and 36.4% of transplants post-policy. Additionally, Trilles et al. have described the transfer of a VCA donor to the transplant hospital prior to organ procurement, allowing the transplant team to minimize CIT by performing the donor recovery and VCA transplant procedures in adjacent operating rooms (17). This case illustrates that allocation distance (distance from donor hospital to transplant hospital) and CIT are not necessarily correlated. The OPTN does not collect data on donor recovery location and we were unable to assess the frequency of donor transfer prior to recovery in our cohort. Fourth, we were unable to assess the policy's impact on VCA non-use (organs recovered for transplant that are not transplanted). VCA allocation was recently integrated into UNet^SM^, the software system that has been used for allocation and management of transplant data for all other solid organs since 1999 (18–21). Prior to this integration, the OPTN did not systematically collect information about the disposition of VCA organs that were offered but not transplanted. Although we were unable to assess VCA non-use for the cohort in this analysis, calculating non-use rates will be possible in future analyses using post-14 September 2023 data.

In conclusion, while results must be interpreted within the context of low VCA transplant volumes and timing of the allocation change relative to the COVID-19 pandemic, data in the 3 years post-implementation suggest that 500 NM circles were a reasonable replacement for OPTN region in VCA allocation. The OPTN will continue to review data to monitor the policy's impact and inform future changes to VCA allocation.

## Data Availability

The data analyzed in this study are subject to the following licenses/restrictions: OPTN data are available upon request to the OPTN. Requests to access these datasets should be directed to https://optn.transplant.hrsa.gov/data/view-data-reports/request-data/.
